# Interactions with DNA Models of the Oxaliplatin Analog (*cis*-1,3-DACH)PtCl_2_ †

**DOI:** 10.3390/ijms25137392

**Published:** 2024-07-05

**Authors:** Alessandra Barbanente, Paride Papadia, Anna Maria Di Cosola, Concetta Pacifico, Giovanni Natile, James D. Hoeschele, Nicola Margiotta

**Affiliations:** 1 Dipartimento di Chimica, Università degli Studi di Bari Aldo Moro, Via E. Orabona 4, 70125 Bari, Italy; 2 Department of Biological and Environmental Sciences and Technologies (DiSTeBA), University of Salento, 73100 Lecce, Italy; 3 Department of Chemistry, Eastern Michigan University, Ypsilanti, MI 48197, USA

**Keywords:** oxaliplatin, cisplatin, *cis*-1,3-diaminocyclohexane, antitumor compounds, DNA adducts

## Abstract

It is generally accepted that adjacent guanine residues in DNA are the primary target for platinum antitumor drugs and that differences in the conformations of the Pt-DNA adducts can play a role in their antitumor activity. In this study, we investigated the effect of the carrier ligand *cis*-1,3-diaminocyclohexane (*cis*-1,3-DACH) upon formation, stability, and stereochemistry of the (*cis*-1,3-DACH)PtG_2_ and (*cis*-1,3-DACH)Pt(d(GpG)) adducts (G = 9-EthlyGuanine, guanosine, 5′- and 3′-guanosine monophosphate; d(GpG) = deoxyguanosil(3′-5′)deoxyguanosine). A peculiar feature of the *cis*-1,3-DACH carrier ligand is the steric bulk of the diamine, which is asymmetric with respect to the Pt-coordination plane. The (*cis*-1,3-DACH)Pt(5′GMP)_2_ and (*cis*-1,3-DACH)Pt(3′GMP)_2_ adducts show preference for the ΛHT and ∆HT conformations, respectively (HT stands for Head-to-Tail). Moreover, the increased intensity of the circular dichroism signals in the *cis*-1,3-DACH derivatives with respect to the analogous *cis*-(NH_3_)_2_ species could be a consequence of the greater bite angle of the *cis*-1,3-DACH carrier ligand with respect to *cis*-(NH_3_)_2_. Finally, the (*cis*-1,3-DACH)Pt(d(GpG)) adduct is present in two isomeric forms, each one giving a pair of H8 resonances linked by a NOE cross peak. The two isomers were formed in comparable amounts and had a dominance of the HH conformer but with some contribution of the ΔHT conformer which is related to the HH conformer by having the 3′-G base flipped with respect to the 5′-G residue.

## 1. Introduction

**Scheme 1:**
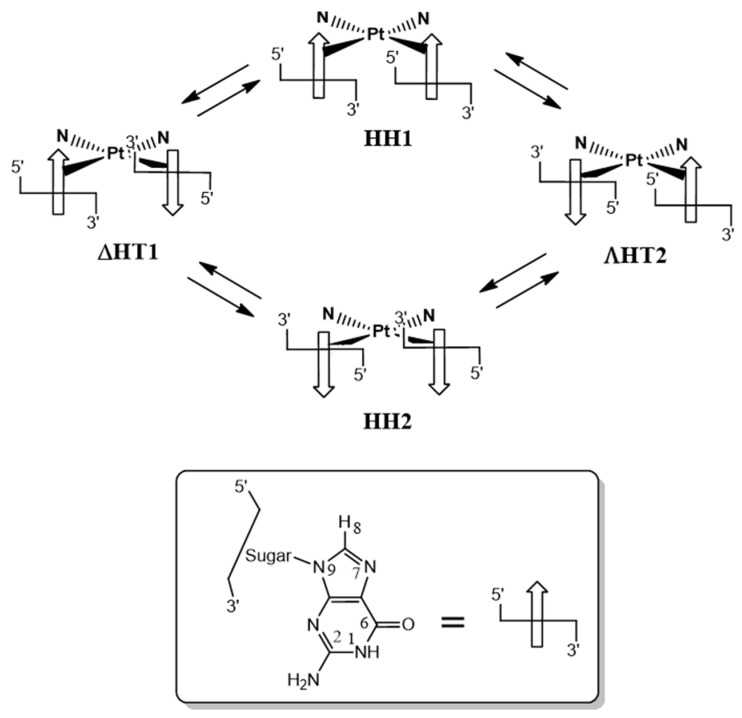
Possible conformers (rotamers) of *cis*-(diam(m)ine)PtG_2_ adducts. The arrows represent the G bases, with their tip symbolizing the hydrogen atom in position 8. In the Head-to-Head (HH) arrangement, both G residues have their H8 atoms on the same side of the Pt coordination plane, while in the Head-to-Tail (HT) arrangement, the two Gs residues have their H8 atoms on opposite sides of the Pt coordination plane. In the latter case, the adduct is asymmetric and can have Δ or Λ chirality. Interconversion between conformers is possible via rotation about the Pt–G bond. In the case of fast rotation on the NMR time scale, only one H8G signal will be observed in the case of G = EtG, while two H8G signals will be observed in the case of G = guanosine/guanotide (see following discussion).

Cisplatin is one of the best-performing antitumor drugs in clinical use [1,2]. It is highly effective in the treatment of testicular and ovarian cancer and is also used, in association with other antitumor drugs, in the treatment of bronchogenic carcinoma, cervical carcinoma, osteosarcoma, melanoma, and neuroblastoma [3]. Cisplatin mainly targets DNA by binding to N7 of adjacent purines of the same strand and forming the so-called 1,2-intrastrand cross-links [4,5,6,7,8]. The two cross-linked guanine bases adopt primarily a Head-to-Head (HH) arrangement, with both G residues having their H8 atoms on the same side of the platinum coordination plane and maintaining the anti conformation of the nucleotides typical of B-DNA (HH1 or HH2 in Scheme 1) [6,9,10,11,12,13,14,15]. In contrast, in the interstrand cross-links (when the two Gs are on opposite strands of DNA), the guanine bases adopt a Head-to-Tail (HT) arrangement [16,17,18,19] with the two H8s on opposite sides of the coordination plane. Intrastrand adducts are thought to be the lesions responsible for the cascade of events that leads to cell death, although interstrand cross-links could also contribute to the anticancer activity [20].

The clinical use of cisplatin is limited by undesirable side effects, including ototoxicity, nephrotoxicity, neurotoxicity, and myelosuppression [21,22]. These drawbacks have stimulated the development of new platinum compounds and the investigation of their mechanism of action. Oxalilplatin, [(1*R*,2*R*)-diaminocyclohexane]oxalatoplatinum(II), has proven to be a valid alternative to cisplatin, as it is active against cisplatin-resistant tumors and is better tolerated in the body. Oxalilplatin contains 1*R*,2*R*-diaminocyclohexane (1*R*,2*R*-DACH) as a carrier ligand; it forms fewer cross-links than cisplatin at equimolar concentrations, is bulkier and more hydrophobic than cisplatin, and the overall pharmacological effects in the cells are different [23,24]. An isomer of 1*R*,2*R*-DACH, the *cis*-1,4-DACH carrier ligand, was recently introduced into platinum-based drugs. In particular, the complex [PtCl_2_(*cis*-1,4-DACH)] (kiteplatin) was extensively investigated by some of us and has since emerged as a drug with very promising anticancer activity. In fact, it was found to have better activity than cisplatin in most cisplatin-resistant cell lines, such as the cisplatin-resistant ovarian C13* cells, and it is also active in the oxalilplatin-resistant colon LoVo-OXP cell line. Moreover, in vivo experiments have also shown that kiteplatin has better activity than cisplatin against platinum-resistant murine leukemias [25,26,27,28,29,30]. In order to unravel the factors responsible for the markedly different pharmacological activity of kiteplatin in comparison to cisplatin and oxaliplatin, the formation, stability, and stereochemistry of the (*cis*-1,4-DACH)PtG_2_ (G = 3′GMP, 5′GMP) [25] and (*cis*-1,4-DACH)Pt(ss-oligo) (ss-oligo = d(GpG), d(GGTTT) and d(TGGT)) [31] adducts were investigated by employing ^1^H and ^31^P 1D and 2D NMR spectroscopy complemented with a combination of molecular mechanism and semi-empirical quantum-chemical calculations [32]. Ranaldo et al. showed, for the first time, that in (*cis*-1,4-DACH)Pt(5′GMP)_2_, by lowering the temperature, the single H8 NMR signal observed at room temperature de-coalesces into four signals arising from the three possible conformers, HH, ΛHT, and ∆HT, with a composition of 33, 51, and 16%, respectively (the carrier ligand *cis*-1,4-DACH is symmetric with respect to the Pt-coordination plane, which renders the two HH conformers of Scheme 1 equivalent) [25]. Different from the previous cases, in (*cis*-1,4-DACH)Pt(d(GpG)) and (*cis*-1,4-DACH)Pt(d(GGTTT)) adducts, the two guanines are crosslinked by the sugar–phosphate backbone with one guanine at the 5′ side and the other guanine at the 3′ side. Also in this case, each guanine (either the 5′G or the 3′G) can rotate about the Pt–N7 bond (such a rotation being accompanied, in general, by change in anti/syn conformation at the corresponding N9-C1′ glycosidic bond) and formation of different rotamers (of the same kind as those shown in Scheme 1). It was found in both cases that the equilibrium between the HH1 and ∆HT1 conformers (present in comparable amounts at 40 °C) shifts towards the more stable HH1 conformer at 0 °C. Notably, with a different oligo ((*cis*-1,4-DACH)Pt(d(TGGT))), the HH1 conformer always becomes dominant, even at high temperatures [31]. An interesting feature of the *cis*-1,4-DACH ligand, absent in previous modeling studies, is the large N-Pt-N bite angle (≥97°) [25], which is expected to slow down the interconversion between possible conformers.

**Scheme 2:**
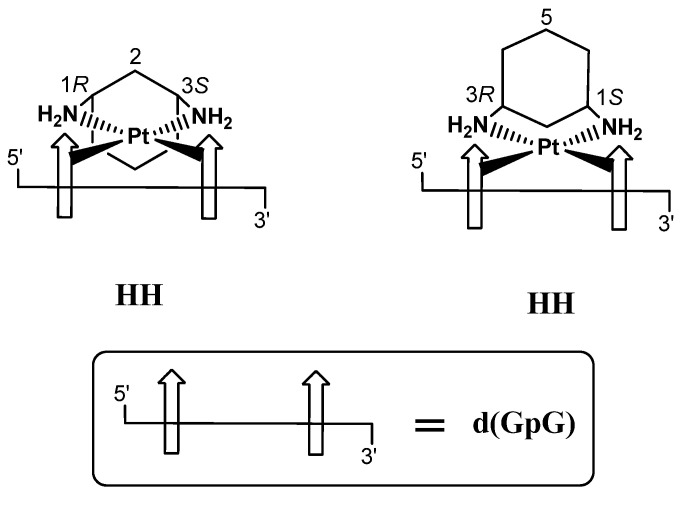
Possible HH adducts obtainable for (*cis*-1,3-DACH)Pt(d(GpG)). The arrows represent the G bases with their tip symbolizing the hydrogen atom in position 8.

In the present study, we have extended the investigation to another isomer of DACH, the *cis*-1,3-diaminocyclohexane ligand. In (*cis*-1,3-DACH)PtCl_2_, the N–Pt–N bite angle is about 92.2° (as determined by X-ray diffraction analysis) [33], which is significantly smaller than that of kiteplatin (≥97°) [25], slightly larger than that of cisplatin (ca. 90°), and significantly larger than that of the analogous compound with ethylenediamine (83°) [34] and 1,2-DACH (83.2°, average value of three Pt complexes) [35]. In previous studies, Hoeschele et al. evaluated the role of the cycloalkane ring in the series of [PtCl_2_(*N-N*)] complexes, where *N-N* = *cis*-1,3-diaminocyclobutane, *cis*-1,3-diaminocyclopentane, and *cis*-1,3-diaminocyclohexane (*cis*-1,3-DACH). It was found that [PtCl_2_(*cis*-1,3-DACH)] destroys cancer cells with greater efficacy than the other two 1,3-diaminocycloalkane derivatives, or cisplatin [36]. Here, we analyze the (*cis*-1,3-DACH)PtG_2_ adducts (G = 9-EthlyGuanine, guanosine, 5′-guanosine monophosphate, and 3′-guanosine monophosphate) by multinuclear NMR and circular dichroism with the aim of characterizing the conformers present in solution. We also reinvestigated the (*cis*-1,3-DACH)Pt(d(GpG)) adduct previously investigated by Inagaki and Sawaki [37] and by Cham et al. [33]. In the latter case, because of the asymmetry of the *cis*-1,3-DACH ligand with respect to the Pt-coordination plane, two isomers can be formed, with one having the G on the 5′ side of the phosphodiester backbone (5′G) *trans* to C3S of DACH (and the G on the 3′ side (3′G) *trans* to C1R) and the other having the 5′G *trans* to C1R of DACH (and the 3′G *trans* to C3S) (Scheme 2).

The two isomers cannot interconvert without breaking the Pt–G bonds (or the Pt–N bonds of the diamine) and can therefore be separated by analytical techniques. Indeed, Cham et al. [33] separated the two isomers, which were formed in a 1:1 ratio, by HPLC. Each of these isomers can form different conformers (of the type shown in Scheme 1) by rotation of individual Gs (either the 5′G or the 3′G) around the Pt–N7 bonds. The lack of intramolecular NOESY or ROESY cross-peaks in the NMR spectra made the determination of which isomer had the 5′G *trans* to C3S, and which one had the 5′G *trans* to C1R impossible. In this study we have been able to, by 2D NOESY experiments, determine that in both isomers, there is a dominance of the HH1 rotamer with a significant contribution of the ∆HT rotamer.

## 2. Results and Discussion

### 2.1. Synthesis and Characterization of [PtCl_2_(cis-1,3-DACH)]

We followed the classical Dhara’s procedure with some modifications [38]. Two intermediates, [PtI_2_(*cis*-1,3-DACH)] and [Pt(OSO_3_)(OH_2_)(*cis*-1,3-DACH)], were prepared. [PtCl_2_(*cis*-1,3-DACH)] was fully characterized by multinuclear 1D and COSY 2D NMR spectroscopy. In the 2D COSY spectrum (ESI, Figure S1b), a cross-peak (A) correlates the two broad doublets at 5.32 and 4.41 ppm that were assigned to the aminic protons NHb and NHa, respectively. The cross-peak (B) observed between the signal falling at 5.32 ppm and at 2.52 ppm (this latter overlapping with the solvent signal) allows to assign the latter signal to the methinic protons H_1/3_. The cross-peaks falling at 4.20/1.54 (C) and 4.20/1.39 (D) ppm allow us to assign the resonances at 4.20, 1.54, and 1.39 ppm to H_5ax_, H_5eq_, and H_4/6_ protons, respectively. The cross-peaks falling at 2.52/1.65 and 2.52/1.46 ppm (E and F, respectively) assign the resonances at 2.52, 1.65, and 1.46 to H_1/3_, H_2eq_, and H_2ax_, respectively. Finally, the cross-peak G correlating the signals at 2.52/1.39 ppm allows us to assign the latter resonance to H_4/6_.

### 2.2. Reaction with G Nucleotides

The (*cis*-1,3-DACH)PtG_2_ adducts were prepared in an NMR tube in acidic D_2_O and then fully characterized by ESI-MS and H^1^ 1D, [^1^H-^13^C]-HSQC, COSY, and NOESY 2D NMR spectroscopy at 300 K.

#### 2.2.1. 9-EthylGuanine (9-EtG)

**Figure 1:**
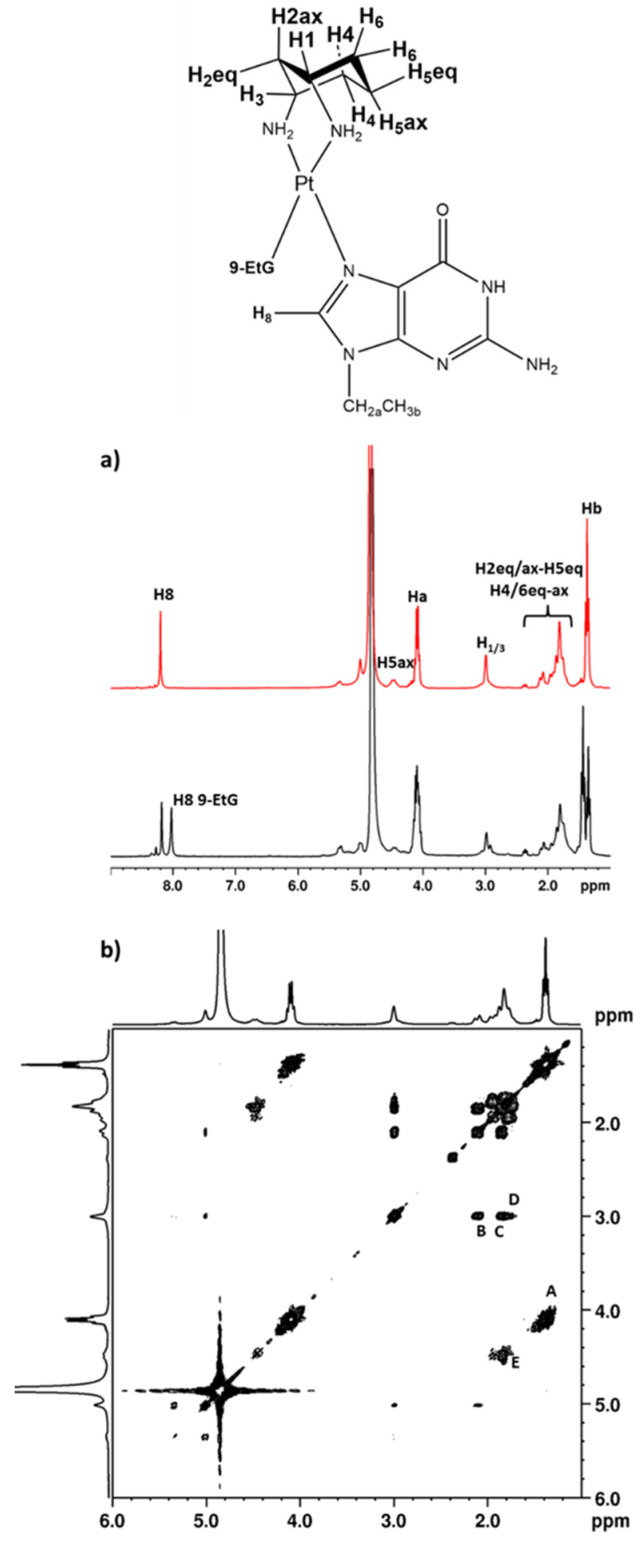
Structure and numbering of atoms of the bis-adduct obtained in the reaction with G = 9-EthylGuanine (9-EtG). (**a**) ^1^H-NMR spectra of the reaction between [Pt(OSO_3_)(OH_2_)(*cis*-1,3-DACH)] and 9-EtG in D_2_O, pH* 3.00: after 2 h (black spectrum) and after three days (red spectrum) at 37 °C; (**b**) COSY spectrum of (*cis*-1,3-DACH)Pt(9-EtG)_2_ adduct in D_2_O.

The ^1^H NMR spectrum of a solution containing [Pt(OSO_3_)(OH_2_)(*cis*-1,3-DACH)] and 9-EtG (molar ratio 1:2; pH*~3), taken soon after mixing of the reagents (Figure 1a, bottom spectrum), exhibited three main signals in the range typical for H8 peaks: the signals at 8.03 and 8.21 ppm were assigned to H8 in free 9-EtG and the bis-adduct, respectively. The small peak falling at ca. 8.3 ppm is assignable to the initially formed mono-adduct, having only one 9-EtG coordinated to platinum. The ^1^H NMR spectrum recorded after a reaction time of 3 days (Figure 1a, top spectrum) exhibited only the H8 signal belonging to the bis-adduct, indicating that the reaction had reached completion. Only one set of G signals was observed for the bis-adduct, indicating that interconversion between the possible conformers (HH and HT) at room temperature was fast on the NMR time scale. The assignment of proton resonances in (*cis*-1,3-DACH)Pt(9-EtG)_2_ was made using a combination of the 1D and 2D NMR methods (chemical shifts are reported in Table 1). In the COSY spectrum (Figure 1b), the cross-peak A observed between the signals falling at 4.10 and 1.38 ppm allowed for the assignment of H_a_ and H_b_ protons of the 9-EtG ethyl group, while the cross peaks at 2.99/2.10 (B), 2.99/1.87 (C), and 2.99/1.81 ppm (D) allowed for the assignment of H_2eq_, H_2ax_, and H_4/6_ protons, respectively. Finally, the broad cross-peak E correlating the signals at 4.49/1.95 ppm and 1.87/1.81 ppm assign the four resonances, in the given order, to H_5ax_, H_5eq_, H_2ax_, and H_4/6_.

#### 2.2.2. Guanosine (Guo)

**Figure 2:**
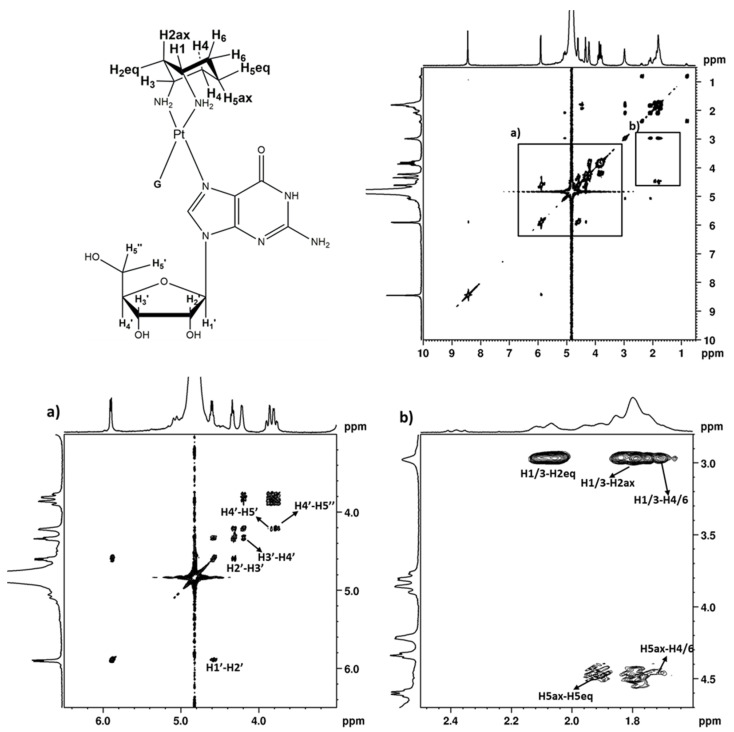
COSY spectrum of (*cis*-1,3-DACH)Pt(Guo)_2_ adduct in D_2_O. (**a**,**b**) expansions of the boxes in the COSY spectrum.

The ^1^H NMR spectrum of a solution containing [Pt(OSO_3_)(OH_2_)(*cis*-1,3-DACH)] and guanosine (molar ratio of 1:2; pH*~3) acquired soon after mixing of the reagents (ESI, Figure S2, bottom spectrum) exhibited three main signals in the range typical for H8 peaks, 8.23, 8.44, and 8.50 ppm; these signals could be assigned to free guanosine, the bis-adduct (*cis*-1,3-DACH)Pt(Guo)_2_, and the mono-adduct (*cis*-1,3-DACH)Pt(OSO_3_)(Guo), respectively. After 3 days, the reaction had reached completion and the ^1^H NMR spectrum (ESI, Figure S2, top spectrum) exhibited only the signal belonging to the bis-adduct (8.44 ppm). The observation of only one H8 signal indicates that the interconversion among the four possible conformers (two HH and two HT) is fast on the NMR time scale at room temperature and that the separation between the signals of the two Gs (expected owing to the asymmetry of the sugar residues) is negligibly small. The assignment of proton resonances in (*cis*-1,3-DACH)Pt(Guo)_2_ was made using a combination of the 1D and 2D NMR methods (chemical shifts are reported in Table 1). The COSY spectrum in D_2_O (Figure 2) showed a cross-peak between the signals at 5.89 and 4.60 ppm. The latter had a cross-peak with a signal at 4.33 ppm which, in turn, had another cross-peak with the signal at 4.23 ppm. Finally, two cross peaks at 4.23/3.84 and 4.23/3.77 were observed. These data allow us to assign the signals at 5.89, 4.60, 4.33, 4.23, 3.84, and 3.77 ppm to H1′, H2′, H3′, H4′, H5′, and H5″ sugar protons, respectively. Moreover, the COSY spectrum showed a cross-peak at 4.46/1.92 ppm assigned to H_5ax_ and H_5eq_ of the *cis*-1,3-DACH moiety. In addition, the signal at 4.46 ppm had a cross-peak with the signal falling at 1.74 ppm, that allowed us to assign the latter signal to the H_4/6_ protons. Finally, the cross peaks at 2.97/2.08, 2.97/1.80, and 2.97/1.74 ppm correlate the methinic protons H_1/3_ with H_2eq_, H_2ax_, and H_4/6_, respectively.

#### 2.2.3. 5′GMP

The ^1^H NMR spectrum of a solution containing [PtCl_2_(*cis*-1,3-DACH)] and 5′GMP (molar ratio 1:2; pH*~3), acquired 24 h after mixing of the reagents (ESI, Figure S3a), exhibited three main signals in the range typical for H8 protons: 8.21, 8.52, and 8.50 ppm, where the first is assigned to H8 of the free 5′GMP and the others to the bis-adduct (*cis*-1,3-DACH)Pt(5′GMP)_2_, respectively. The ^1^H NMR spectrum recorded after 6 days (ESI, Figure S3b) exhibited only the two signals belonging to the bis-adduct, which was the only product present in solution as confirmed by ESI–MS, indicating that the reaction had reached completion. As in the case of 9-EtG and guanosine, the interconversion between possible conformers at room temperature was fast on the NMR time scale (Scheme 1); however, unlike the case of the guanosine derivative previously investigated, it is now possible to detect two signals for the H8 protons stemming from the presence of the asymmetric sugar–phosphate substituent that renders the two G residues non-equivalent even in the case of fast interconversion between conformers.

**Figure 3:**
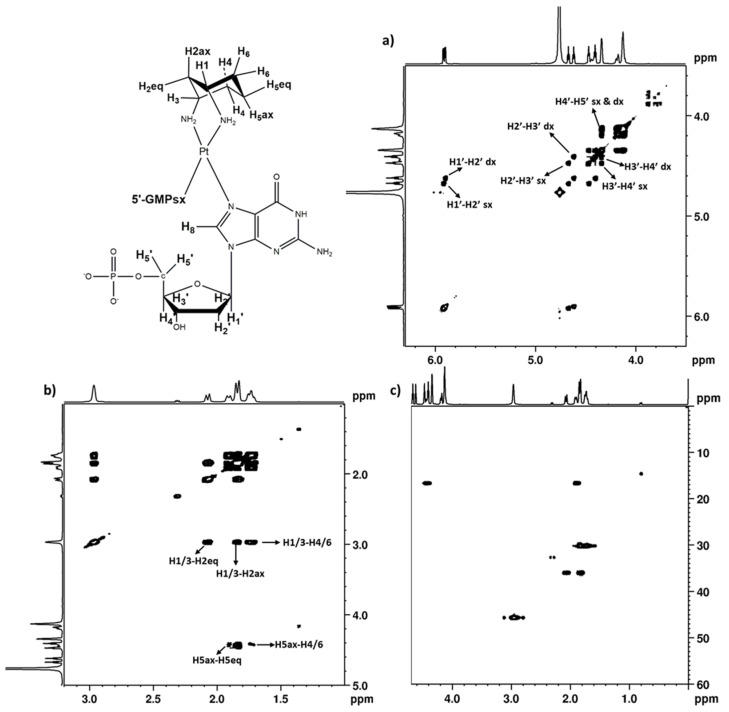
Expansions of the 2D COSY (**a**,**b**) and [^1^H-^13^C]-HSQC (**c**) spectra of (*cis*-1,3-DACH)Pt(5′GMP)_2_ in D_2_O.

The assignment of proton resonances in (*cis*-1,3-DACH)Pt(5′GMP)_2_ was made using a combination of 1D and 2D NMR methods (COSY, NOESY, [^1^H,^13^C] HSQC; Table 1). The COSY spectrum in D_2_O (Figure 3a) showed a cross-peak between signals at 5.92 and 4.67 ppm. The latter had a cross-peak with a signal at 4.47 ppm which, in turn, was correlated with the signal at 4.34 ppm. Finally, another cross-peak correlated the signals at 4.34 and 4.13 ppm. These data allow us to assign the signals at 5.92, 4.97, 4.47, 4.34, and 4.13 ppm to H1′, H2′, H3′, H4′, and H5′ sugar protons of one 5′GMP. Similarly, the set of signals at 5.90, 4.62, 4.41, 4.34, and 4.13 ppm were assigned to H1′, H2′, H3′, H4′, and H5′ sugar protons of the second 5′GMP. These differences in the chemical shifts of the two 5′GMP reflect their non-equivalence. The assignment of the proton resonances belonging to the *cis*-1,3-DACH is based on the COSY spectrum (Figure 3b). A cross-peak at 4.44/1.91 ppm assigns these resonances to H_5ax_ and H_5eq_ (^2^J_H-H_ = 13.93 Hz). The signal at 4.44 ppm has a cross-peak with the signals falling at 1.73 ppm, that allows to assign the H_4/6_ protons. Finally, the cross-peaks at 2.97/2.07, 2.97/1.84, and 2.97/1.73 ppm correlate the methinic protons H_1/3_ with H_2eq_, H_2ax_, and H_4/6_, respectively. The assignment of the signal at 2.97 ppm to H_1/3_ was also confirmed by the [^1^H-^13^C]-HSQC spectrum (Figure 3c), that assigned the signal at 45.70 ppm to C_1/3_.

**Figure 4:**
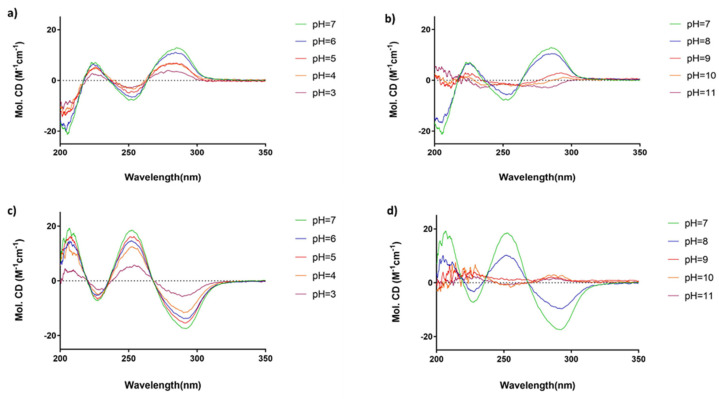
CD spectra of (*cis*-1,3-DACH)Pt(5′GMP)_2_ (**a**,**b**) and (*cis*-1,3-DACH)Pt(3′GMP)_2_ (**c**,**d**) in solution: (**a**,**c**) pH = 7–3; (**b**,**d**) pH = 7–11.

Although the interconversion between conformers is fast on the NMR time scale, it is possible to detect which conformer is dominant in solution using CD spectroscopy. The CD spectrum of the bis-adduct (*cis*-1,3-DACH)Pt(5′GMP)_2_ (Figure 4) showed two positive Cotton effects (226 and 285 nm) and two negative Cotton effects (205 and 254 nm) that indicate that the dominant conformer in solution has ΛHT conformation. This result is in agreement with previous studies [15,25], indicating that the ΛHT conformer is stabilized by the possible interactions of the 5′-phosphate of each G with the N1H group of the other G. By recording the CD spectra at different pH values, it was observed that the intensity of the CD signals increased from pH 3 to pH 7 (Figure 4a), whereas it decreased from pH 7 to pH 11 (Figure 4b). Thus, the highest intensity of the CD signals was observed at neutral pH, where the 5′-phosphate group is completely deprotonated and can interact with the N1H group of the cis G that has not yet undergone deprotonation.

#### 2.2.4. 3′GMP

**Figure 5:**
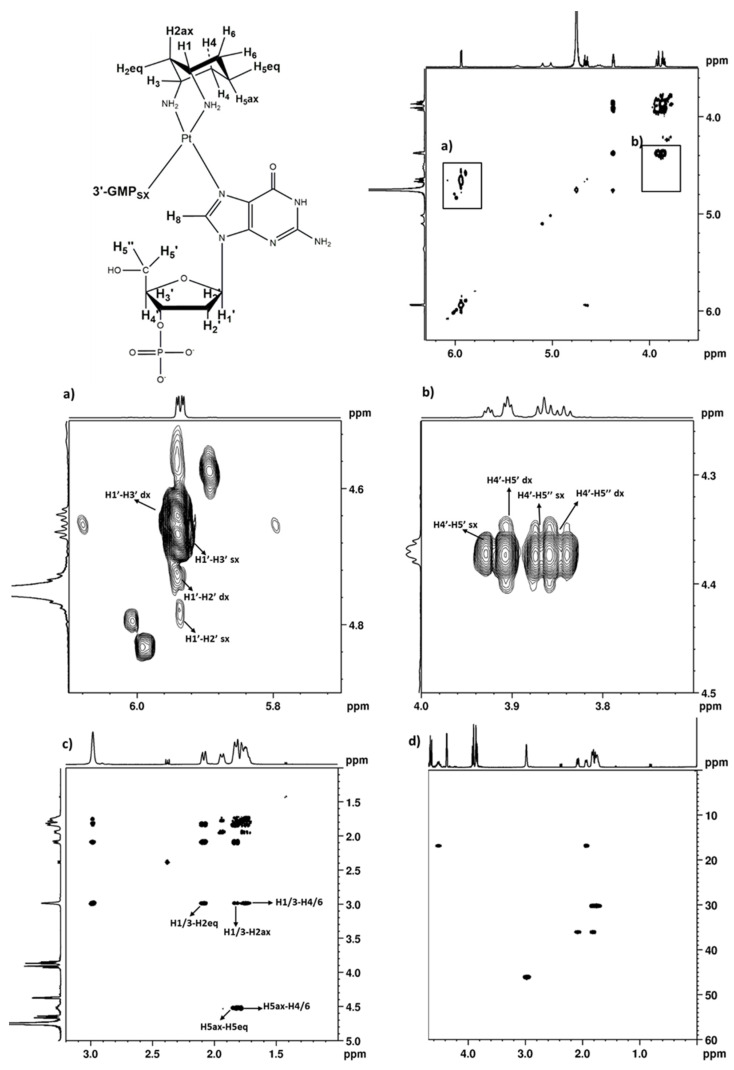
Top right: COSY spectrum of (*cis*-1,3-DACH)Pt(3′GMP)_2_ in D_2_O. (**a**–**c**) expansions of the COSY spectrum. (**d**) [^1^H-^13^C]-HSQC of (*cis*-1,3-DACH)Pt(3′GMP)_2_ in D_2_O.

The ^1^H NMR spectrum of a solution containing [PtCl_2_(*cis*-1,3-DACH)] and 3′GMP (molar ratio 1:2; pH*~3) acquired 48 h after mixing of the reagents (ESI, Figure S4, bottom spectrum) exhibited three main signals in the range typical for H8 peaks: 8.13, 8.47, and 8.49 ppm, where the first was assigned to H8 of free 3′GMP and the others were assigned to the bis-adduct (*cis*-1,3-DACH)Pt(3′GMP)_2_. The ^1^H NMR spectrum recorded after 13 days (ESI, Figure S4, top spectrum) exhibited only the two signals belonging to the bis-adduct, as further confirmed by ESI–MS, indicating that the reaction had reached completion. The assignment of proton resonances in (*cis*-1,3-DACH)Pt(3′GMP)_2_ was made using a combination of 1D and 2D NMR methods (COSY, NOESY, [^1^H,^13^C] HSQC; Table 1 and Figure 5).

In order to detect which conformer is dominant in solution, CD spectroscopy was used. The CD spectrum of (*cis*-1,3-DACH)Pt(3′-GMP)_2_ (Figure 4c,d) showed two negative Cotton effects (228 and 292 nm) and two positive Cotton effects (206 and 252 nm) that are indicative of dominant HT conformer with ∆ chirality. Also, in this case, the CD spectrum was recorded at different pH values. By increasing the pH from 3.0 to 7.0 (Figure 4c), the intensities of the CD signals increased. Conversely, a further increase in the pH from 7.0 to 11.0 (Figure 4d) caused the intensities of the CD signals to decrease. The literature data indicate that in the Pt-adducts with cis 3′GMP ligands, the ∆HT conformer is stabilized by H-bonding interactions between the phosphate group of one nucleotide and the N1H group of the other nucleotide. This interaction is greatest at pH 7, where the phosphate group is completely deprotonated while the N1H group has not yet started to undergo deprotonation [15].

#### 2.2.5. Variable Temperature NMR Experiments on (*cis*-1,3-DACH)Pt(5′GMP)_2_ and (*cis*-1,3-DACH)Pt(3′GMP)_2_

As in the case of cisplatin and analogous compounds with primary chelating diamines (such as ethylenediamine and 1,2-DACH), and also in the case of (*cis*-1,3-DACH)PtG_2_ adducts, the interconversion between conformers is fast on the NMR time scale at room temperature. The rate of interconversion can slow down by decreasing the temperature; therefore, the solutions of (*cis*-1,3-DACH)Pt(5′GMP)_2_ and (*cis*-1,3-DACH)Pt(3′GMP)_2_ in 2:1 (*v*/*v*) D_2_O/CD_3_OD (pH* = 5.83) were subjected to ^1^H NMR detection over the temperature range 298–278 K. The G H8 NMR signals, located in an isolated region of the spectrum, underwent a broadening accompanied by a downfield shift and an increase in separation between the two H8 signals, indicative of the increasing percentage of HH conformer (ESI, Figure S5). However, even at the lowest temperature investigated (278 K), the interconversion between possible conformers was still fast on the NMR time scale and only an average signal was observed for the H8 proton of each nucleotide. This finding implies that the barriers to rotation of the two Gs in (*cis*-1,3-DACH)PtG_2_ adducts is very low. This could be related to the value of the N–Pt–N bite angle of the diamine (about 92.2°, similar to that found in cisplatin), which is significantly smaller than that of kiteplatin (bite angle of *cis*-1,4-DACH ≥ 97°). Therefore, only in the case of (*cis*-1,4-DACH)Pt(5′GMP)_2_, by lowering the temperature, it was possible to slow down the rate of interconversion between conformers to the extent that the ^1^H NMR spectrum could show three separate sets of signals belonging to the three possible conformers (two HT and one HH being the HH1 and HH2 equivalents) [25].

#### 2.2.6. Deoxyguanosil(3′-5′)deoxyguanosine (d(GpG))

**Figure 6:**
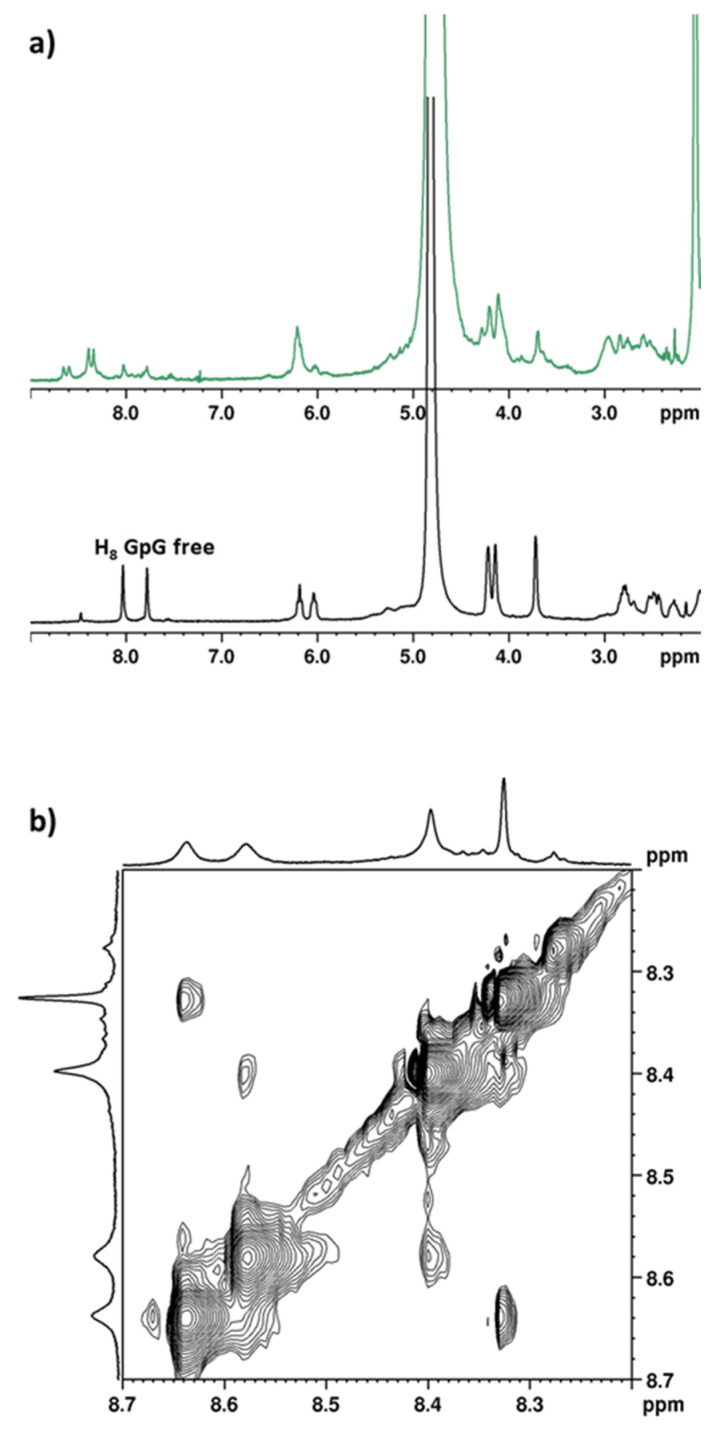
(**a**) ^1^H-NMR spectra of the reaction between [Pt(OSO_3_)(OH_2_)(*cis*-1,3-DACH)] and d(GpG) in D_2_O, pH* 3.00: soon after mixing of the reagents (black) and after one day (green) at 37 °C; (**b**) Expansion of the NOESY spectrum of (*cis*-1,3-DACH)Pt(d(GpG)) recorded after completion of the reaction.

A solution containing [Pt(OSO_3_)(*cis*-1,3-DACH)(OH_2_)] and d(GpG) (molar ratio 1:1; pH*~3) was monitored over time by ^1^H NMR spectroscopy (Figure 6a). After 24 h, the pair of H8 signals of free d(GpG) (8.02 and 7.67 ppm) completely disappeared; meanwhile, two new pairs of H8 signals appeared, which were shifted downfield and quite broad. This is consistent with the formation of the two expected isomers for (*cis*-1,3-DACH)Pt(d(GpG)) (as shown in Scheme 2 and already reported by Inagaki and Sawaki [37] and by Tsuey Cham et al. [33]). The NOESY spectrum (Figure 6b) showed the presence of NOE cross-peaks between the H8 signals at 8.39 and 8.57 ppm and between the signals at 8.33 and 8.64 ppm. The presence of such H8–H8 cross-peaks assign the pairs of signals 8.39/8.57 and 8.33/8.64 to the two isomers, both of which must have a major component of HH conformer that is responsible for the H8/H8 NOE. Unfortunately, no cross-peaks could be detected between the guanine H8 and the protons of *cis*-1,3-DACH ligand (particularly H2eq or H5eq) which would allow us to assign the configuration to each isomer. This is likely a consequence of the dynamic nature of the two coordinated guanines (contribution of different rotamers) and of the *cis*-1,3-DACH ligand (chair and boat conformations of the cyclohexane ring), which brought the H2eq–H8 and H5eq–H8 distances outside the limits of detection of NOE cross-peaks.

It is well known that the major adduct formed by cisplatin with DNA is an intrastrand d(GpG) cross-link with the two guanine bases in the HH conformation, and that the presence of carrier ligands different from ammines may affect biodistribution, rate, and type of DNA adduct formation [7,15]. The antitumor activity of Pt-drugs is mediated by different cellular proteins (mismatch-repair and damage-recognition proteins such as high-mobility group box protein 1 (HMGB1)), TATA box-binding protein, and human upstream binding factor) that specifically recognize DNA adducts formed by these drugs [2,3,39,40,41,42]. Therefore, the shape and bulk of the carrier ligand, as it projects out away from the DNA helix, is likely to influence the interaction with nucleic acid binding proteins or repair enzymes thus impacting the antitumor activity. Our data obtained with d(GpG) confirm that the Pt(*cis*-1,3-DACH) residue is also capable of forming two HH adducts. The data obtained in vitro by Hoeschele and colleagues on a panel of four tumor cell lines indicate that [PtCl_2_(*cis*-1,3-DACH)] displays significantly higher cytotoxicity than cisplatin and comparable or even better activity than kiteplatin and oxaliplatin [36]. We are quite confident that both adducts of [PtCl_2_(*cis*-1,3-DACH)] with DNA can be formed in vivo; however, at this stage of the investigation, it is not possible to predict which of the two adducts can have better antitumor activity.

## 3. Materials and Methods

All starting materials and solvents were purchased from Sigma-Aldrich. ^1^H NMR and ^31^P NMR spectra were recorded on Bruker Avance DPX 300 MHz instrument (Bruker Italia, Milano, Italy). 2D COSY, NOESY, and [^1^H-^13^C]-HQSC spectra were recorded on Bruker Avance III 600 MHz instrument (Bruker Italia, Milano, Italy) equipped with a cryoprobe. Chemical shifts (δ) are given in parts per million and referenced to the internal standard sodium 3-(trimethylsilyl)propionate (TSP). ^1^H NMR experiments at different temperatures were performed using the heating control unit of the spectrometer. CD and UV–vis spectra were recorded on a Jasco J-810 spectropolarimeter (Jasco Europe S.r.l., Milano, Italy) at room temperature over the wavelength range 200–350 nm. The scan rate was 50 nm/min, and data were sampled every 0.1 nm. The path length of the cell was 0.1 cm. CD spectra were processed with the software of the instrument (Spectra Manager Version 1.40.00 Build 2); to the output data, a simple smoothing was applied to reduce noise and solvent blank was then subtracted. The measured original CD data were then converted to molar circular dichroic absorption without applying any Gaussian or Lorentzian correction. A Crison Micro-pH meter (model 2002; Crison, Alella, Barcelona) equipped with Crison standard buffer solutions at pH 4.01, 7.02, and 9.26 was used for pH measurements. Values of pH for D_2_O solutions were indicated as pH* values and were not corrected for the effect of deuterium on the glass electrodes [43]. ESI–MS (electrospray ionization mass spectrometry) experiments were performed with a dual electrospray interface and a quadrupole time-of-flight mass spectrometer (Agilent 6530 Series Accurate-Mass Quadrupole Time-of-Flight (Q-TOF) LC–MS; Agilent, Pavia, Italy).

### 3.1. Synthesis of the Complexes

#### 3.1.1. [PtCl_2_(*cis*-1,3-DACH)]

This complex was prepared following a procedure that contemplates the preparation of two intermediates, [PtI_2_(*cis*-1,3-DACH)] and [Pt(OSO_3_)(OH_2_)(*cis*-1,3-DACH)].

K_2_PtCl_4_ (500 mg, 1.2 mmol) was dissolved in 20 mL of water and treated with 1.6 g of KI (8-fold excess). The reaction mixture was stirred for 5 min at room temperature and treated with 4 mL of a solution containing *cis*-1,3-diaminocyclohexane (0.145 mL, 1.21 mmol). A precipitate formed immediately, and the resulting suspension was stirred at 40 °C for 2.5 h. The dark yellow precipitate was isolated by filtration of the mother liquor, washed with cold water and diethylether, and then dried under vacuum. Yield, 86% (583 mg, 1.035 mmol). Anal. calcd. for [PtI_2_(*cis*-1,3-DACH)] (PtI_2_N_2_C_6_H_14_): C, 12.79; H, 2.51; N, 4.97%. Found: C, 13.06; H, 2.61; N, 4.90%. ESI–MS calcd. for (C_6_H_14_N_2_I_2_PtNa) = 585.8743; found: *m*/*z* 585.8743 [M+Na]^+^. ^1^H-NMR (Acetone-d_6_): 3.25 (2H, CH_1/3_), 2.06 (1H, CH_2eq_), 4.82 (2H, NH_a_), 4.15 (2H, NH_b_), 4.65 (1H, CH_5ax_), 1.90 (1H, CH_5eq_), 1.77 (1H, CH_2ax_), 1.71 (4H, CH_4/6_) ppm. The numbering of protons is analogous to that of [PtCl_2_(*cis*-1,3-DACH)] reported in Figure S1a.

[PtI_2_(*cis*-1,3-DACH)] (300 mg, 0.532 mmol) was dissolved in 20 mL of H_2_O and the solution was treated with Ag_2_SO_4_ (166 mg, 0.532 mmol) and stirred at 40 °C for 18 h in the dark. The suspension was filtered through Celite in order to remove AgI and the solvent was evaporated under reduced pressure, yielding the desired compound as an orange residue. Yield, 81% (182 mg, 0.43 mmol). Anal. calcd. for [Pt(OSO_3_)(OH_2_)(*cis*-1,3-DACH)] (PtN_2_SO_5_C_6_H_14_∙H_2_O): C, 16.30; H, 4.11; N, 6.34%. Found: C, 16.02; H, 3.94; N, 6.12%.

[Pt(OSO_3_)(OH_2_)(*cis*-1,3-DACH)] (147 mg, 0.35 mmol) was dissolved in 75 mL of H_2_O and treated with KCl (337 mg, 4.52 mmol, 13-fold excess). The pH of the reaction mixture was brought to 1–2 with HCl 1.0 M and stirred at 55 °C overnight. The solvent was removed under reduced pressure and the light yellow solid was washed with a small amount of cold water. Yield, 67% (88 mg, 0.23 mmol). Anal. calcd. for [PtCl_2_(*cis*-1,3-DACH)] (PtN_2_Cl_2_C_6_H_14_): C, 18.96; H, 3.71; N, 7.37%. Found: C, 18.89; H, 3.60; N, 7.15%. ESI-MS calcd. for (C_6_H_14_N_2_Cl_2_PtNa) = 403.0030; found: *m*/*z* 403.0041 [M+Na]^+^. ^1^H-NMR (DMSO-d_6_): 2.52 (2H, CH_1/3_), 1.65 (1H, CH_2eq_), 4.41 (2H, NH_a_), 5.32 (2H, NH_b_), 5.20 (1H, CH_5ax_), 1.54 (1H, CH_5eq_), 1.46 (1H, CH_2ax_), 1.39 (4H, CH_4/6_) ppm. The numbering of protons is reported in Figure S1a.

#### 3.1.2. (*cis*-1,3-DACH)PtG_2_ Adducts (G = 9-EthylGuanine, Guanosine, 3′-GMP, and 5′-GMP)

A solution containing G (0.027 mmol) and [PtCl_2_(*cis*-1,3-DACH)] (0.013 mmol) in 1.0 mL of D_2_O was adjusted to pH*~3 with DClO_4_ 0.1 M and transferred into an NMR tube (in the case of G = 9-EtG and Guanosine, [Pt(OSO_3_)(OH_2_)(*cis*-1,3-DACH)] was used instead of [PtCl_2_(*cis*-1,3-DACH)]). The concentration of the platinum complex was 13 mM. The progress of the reaction was monitored by ^1^H- and ^31^P-NMR and the disappearance of the free G H8 signal indicated that the reaction was complete. At the end of the reaction, aliquots of the deuterated mother solutions were used for ESI–MS analyses.

ESI-MS calcd. for (*cis*-1,3-DACH)Pt(9-EtG)_2_ (PtC_20_H_32_N_12_O_2_): 667.2419; found *m*/*z*: 667.2377 [M]^+^. Calcd. for (*cis*-1,3-DACH)Pt(3′-GMP)_2_ (PtC_26_H_42_N_12_O_16_P_2_): 1035.1965; found *m*/*z*: 1035.1918 [M-H]^−^. Calcd. for (*cis*-1,3-DACH)Pt(5′-GMP)_2_ (PtC_26_H_42_N_12_O_16_P_2_): 1035.1965; found *m*/*z*: 1037.2154 [M-H]^−^.

#### 3.1.3. (*cis*-1,3-DACH)Pt(d(GpG)) Adduct (d(GpG) = Deoxyguanosil(3′-5′)deoxyguanosine)

A solution of d(GpG) (0.0047 mmol) and [Pt(OSO_3_)(OH_2_)(*cis*-1,3-DACH)] (0.0047 mmol) in 1 mL of D_2_O was adjusted to pH*~3 with DClO_4_ 0.1 M and then transferred into an NMR tube. The concentration of the platinum complex was 4.7 mM. The progress of the reaction was monitored by ^1^H-NMR, and the disappearance of the free d(GpG) H8 signals indicated that the reaction was complete. At the end of the reaction, aliquots of the deuterated mother solution were used for ESI–MS analyses.

ESI–MS calcd. for (*cis*-1,3-DACH)Pt(d(GpG)) (PtC_26_H_38_N_12_O_10_P): 905.2243; found *m*/*z*: 904.2273 [M-H]^−^.

### 3.2. Solutions for Circular Dichroism (CD) Spectroscopy

Aliquots (25 μL) of the (*cis*-1,3-DACH)Pt(G)_2_ solutions used in the NMR investigations (13 mM) were diluted by addition to an aqueous solution of Na_2_SO_4_ (0.5 mL, 50 mM; the salt required to maintain a constant ionic strength) to a final complex concentration of 5 × 10^−4^ M. The pH of the solutions was adjusted to values in the range 3–11 by addition of H_2_SO_4_ (1.2 × 10^−2^ M) or NaOH (2.5 × 10^−2^ M).

## 4. Conclusions

In the present work, the behavior of the (*cis*-1,3-DACH)PtG_2_ (G = 9-EtG, Guo, 5′GMP, and 3′GMP) and (*cis*-1,3-DACH)Pt(d(GpG)) adducts has been investigated by ^1^H 1D and 2D NMR spectroscopy. The behavior of (*cis*-1,3-DACH)Pt(5′GMP)_2_ and (*cis*-1,3-DACH)Pt(3′GMP)_2_ agrees with the results of previous studies [25] performed on similar adducts of Pt-complexes. In particular, it has been confirmed that when the diam(m)ine carrier ligand is deprived of steric bulk on the coordination plane, the interconversion between possible rotamers is fast on the NMR time scale. Moreover, it has been confirmed by CD spectroscopy that (*cis*-1,3-DACH)Pt(5′GMP)_2_ has a preference for the ΛHT conformer, which allows for the formation of an intramolecular hydrogen bond between the phosphate group of one 5′-GMP and the N1H of the *cis* nucleotide. On the other hand, the (*cis*-1,3-DACH)Pt(3′GMP)_2_ adduct has a preference for the ∆HT conformer, which allows for a hydrogen bond between the 3′-phosphate of one 3′GMP and the N1H of the *cis* 3′GMP. In both the 5′GMP and the 3′GMP adducts, the Cotton effects were the largest at neutral pH, where the phosphate group was completely deprotonated while the N1H had not yet started to undergo deprotonation. By lowering the temperature, the H8 signals underwent a broadening accompanied by a downfield shift and an increase in separation between the two H8 signals. Both effects are indicative of increasing percentage of the HH conformer.

The behavior of the (*cis*-1,3-DACH)Pt(d(GpG)) adduct was also in agreement with previous studies [33,37]. Due to the asymmetry of the *cis*-1,3-DACH ligand with respect to the Pt-coordination plane, two isomers could be formed, leading to two pairs of H8 signals. The assignment of the signals to each pair was possible thanks to the observation of H8–H8 NOE cross-peaks between signals of the same pair. The two isomers were formed in comparable amounts and, despite having a dominance of the HH conformer, there was also a contribution of the ΔHT conformer, which is related to the HH conformer, by having a syn conformation of the 3′-G residue and the base flipped with respect to the 5′-G residue. Such a flipping of the 3′-G residue reflects on the greater broadening of the corresponding H8 signal (the low field signal within each pair).

Future studies will focus on the interaction of the (*cis*-1,3-DACH)Pt moiety with other single and double-stranded oligonucleotides as a further step in the elucidation of the anticancer activity of [PtCl_2_(*cis*-1,3-DACH)].

## Figures and Tables

**Table 1:** ^1^H NMR shifts (ppm) for (*cis*-1,3-DACH)PtG_2_, in D_2_O at room temperature. In the case of (*cis*-1,3-DACH)Pt(5′GMP)_2_ and (*cis*-1,3-DACH)Pt(3′GMP)_2_, the two Gs are not equivalent and two sets of signals are observed.

Adduct	G *sx*/*dx*									*cis*-1,3-DACH					
	H8	CH2a	CH3b	H1′	H2′	H3′	H4′	H5′	H5″	H1/3	H2ax	H2eq	H5ax	H5eq	H4/6
9-EtG	8.21	4.10	1.38	-	-	-	-	-	-	2.99	1.87	2.10	4.49	1.95	1.81
Guanosine	8.44	-	-	5.89	4.60	4.33	4.23	3.84	3.77	2.97	1.80	2.08	4.46	1.92	1.74
5′GMP	8.52	-	-	5.92	4.67	4.47	4.34	4.13	4.13	2.97	1.84	2.07	4.44	1.91	1.73
	8.50	-	-	5.90	4.62	4.41	4.34	4.13	4.13						
3′GMP	8.49	-	-	5.94	4.64	4.37	3.93	3.84	3.84	2.98	1.83	2.08	4.51	1.94	1.75
	8.47	-	-	5.94	4.67	4.37	3.91	3.86	3.86						

## Data Availability

Dataset available on request from the authors.

## Supplementary Materials

The following supporting information can be downloaded at: https://www.mdpi.com/article/10.3390/ijms25137392/s1.
