# Supplementary material for: Interactions with DNA Models of the Oxaliplatin Analog (cis-1,3-DACH)PtCl2
Source: Int J Mol Sci. 2024 Jul 5;25(13):7392. doi: 10.3390/ijms25137392 (PMC11242235; doi:10.3390/ijms25137392)
Supplement: Supplementary file 1 [file ijms-25-07392-s001.zip › ijms-3082438-supplementary.pdf]

Electronic Supplementary Information

Interaction with DNA models of the oxaliplatin analog (*cis*-1,3-DACH)$PtCl_2$.

Alessandra Barbanente[1], Paride Papadia[2], Anna Maria Di Cosola[1], Concetta Pacifico[1], Giovanni Natile[1], James D. Hoeschele[3], and Nicola Margiotta[1].

[1] *Dipartimento di Chimica, Università degli Studi di Bari Aldo Moro, Via E. Orabona 4, 70125 Bari, Italy;*

[2] *Department of Biological and Environmental Sciences and Technologies (DiSTeBA), University of Salento, 73100 Lecce, Italy;*

[3] *Department of Chemistry, Eastern Michigan University, Ypsilanti, MI 48197, USA.*

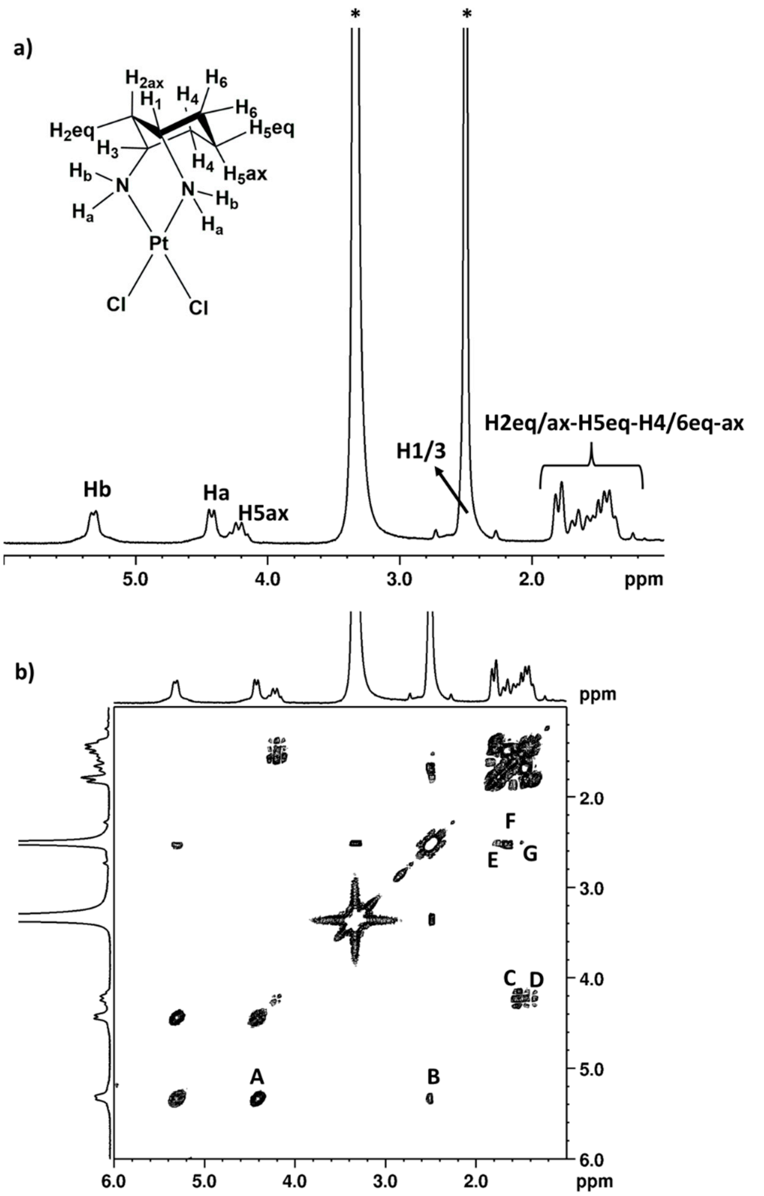

Figure S1. (**a**) ^{1}H NMR and (**b**) 2D COSY spectra of $[PtCl_2(\textit{cis}\text{-}1,3\text{-DACH})]$ in DMSO-d_6.

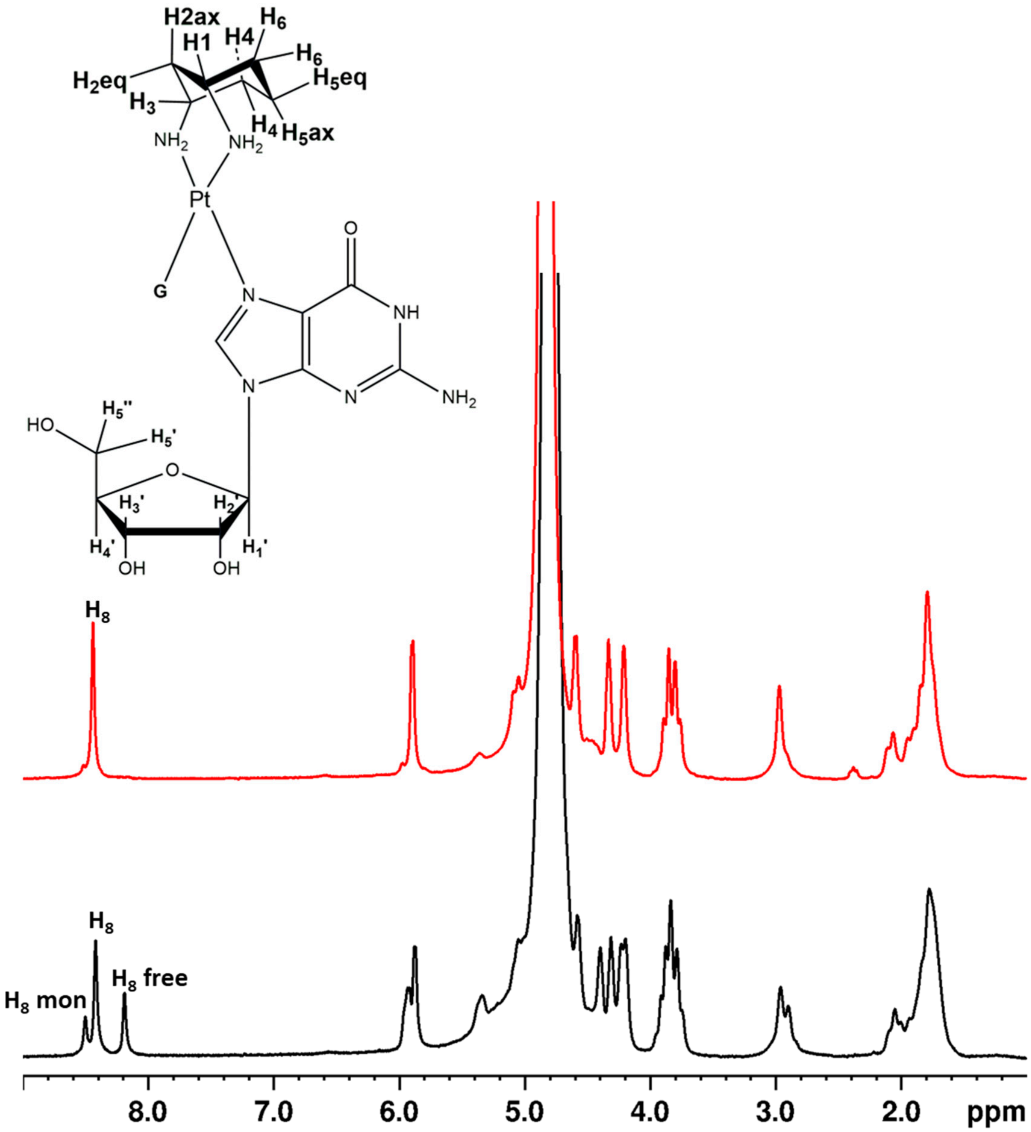

Figure S2. Monitoring of the reaction between [Pt(OSO_3)(OH_2)(*cis*-1,3-DACH)] and Guanosine in D_2O, pH* 3.00. ^{1}H-NMR spectrum registered after 2h (black) and after three days (red) at 37 °C. The signals belong to the H8 of different species are indicated as: H8free for unreacted G, H8mon for the mono-adduct, and H8 for the bis-adduct.

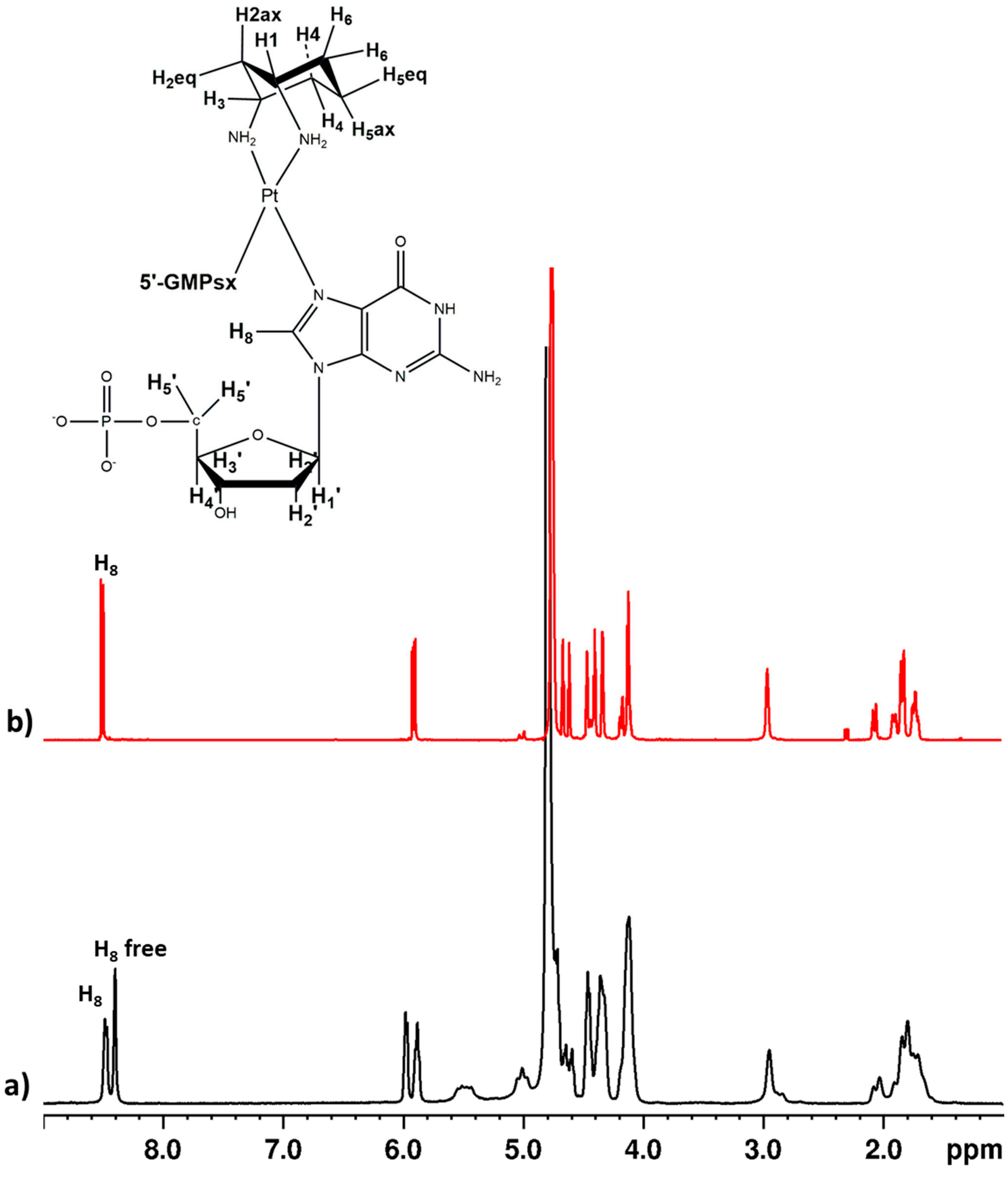

Figure S3. Monitoring of the reaction between [$PtCl_2$(*cis*-1,3-DACH)] and 5'GMP in D_2O, pH* 3.00. ^{1}H-NMR spectrum recorded after 24h (black, a)) and after six days (red, b)) at 37 °C. The signals belonging to the H8 of different species are indicated as: H8free for unreacted 5'GMP, H8 for the two 5'-GMP of the bis-adduct.

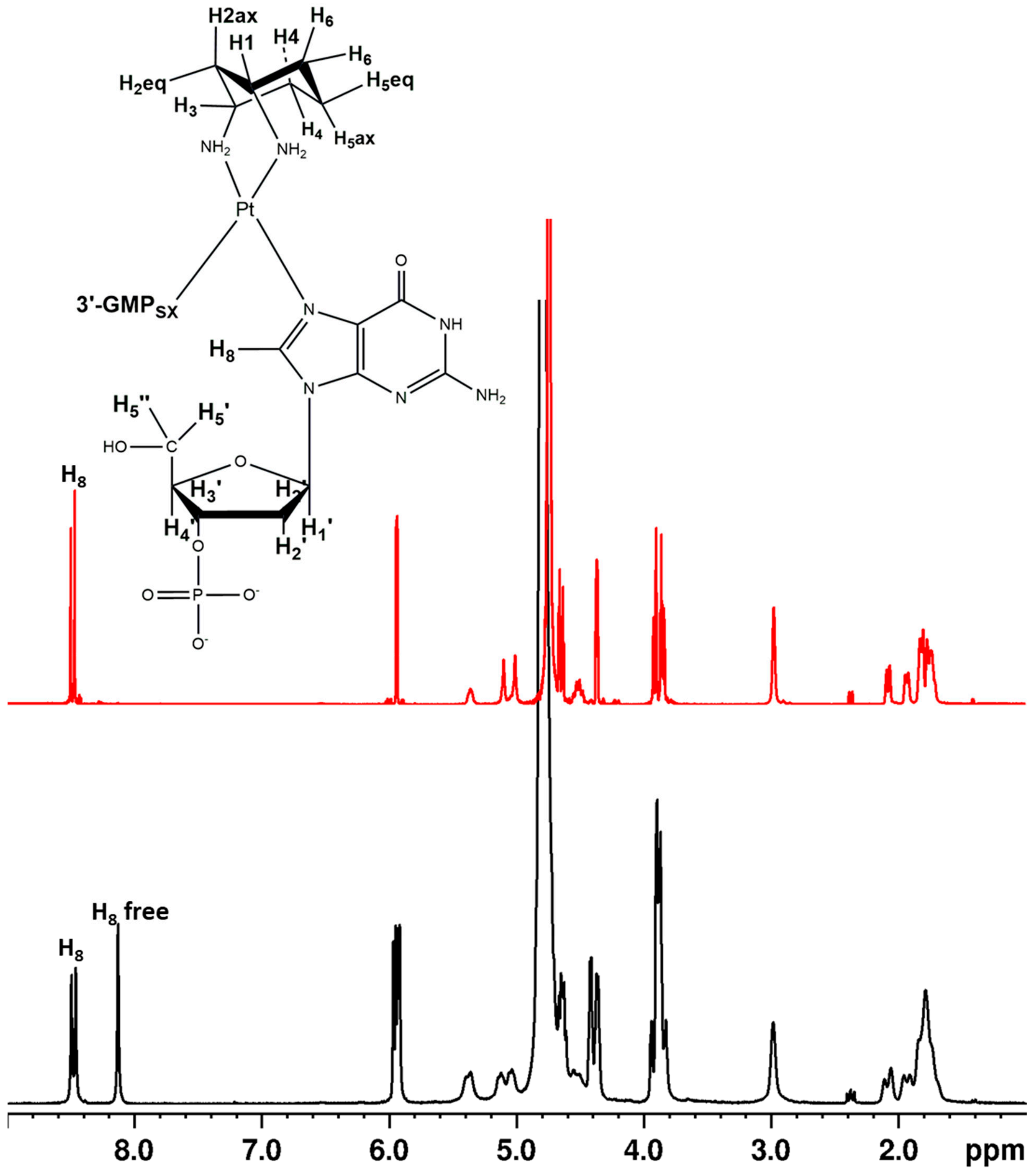

Figure S4: Monitoring of the reaction between [$PtCl_2$(*cis*-1,3-DACH)] and 3'GMP in D_2O, pH* 3.00. ^{1}H-NMR spectrum detected after 48h (black) and 13 days (red) at 37 °C. The signals belonging to the H8 of different species are indicated as: H8free for unreacted 3'GMP, H8 for the bis-adduct.

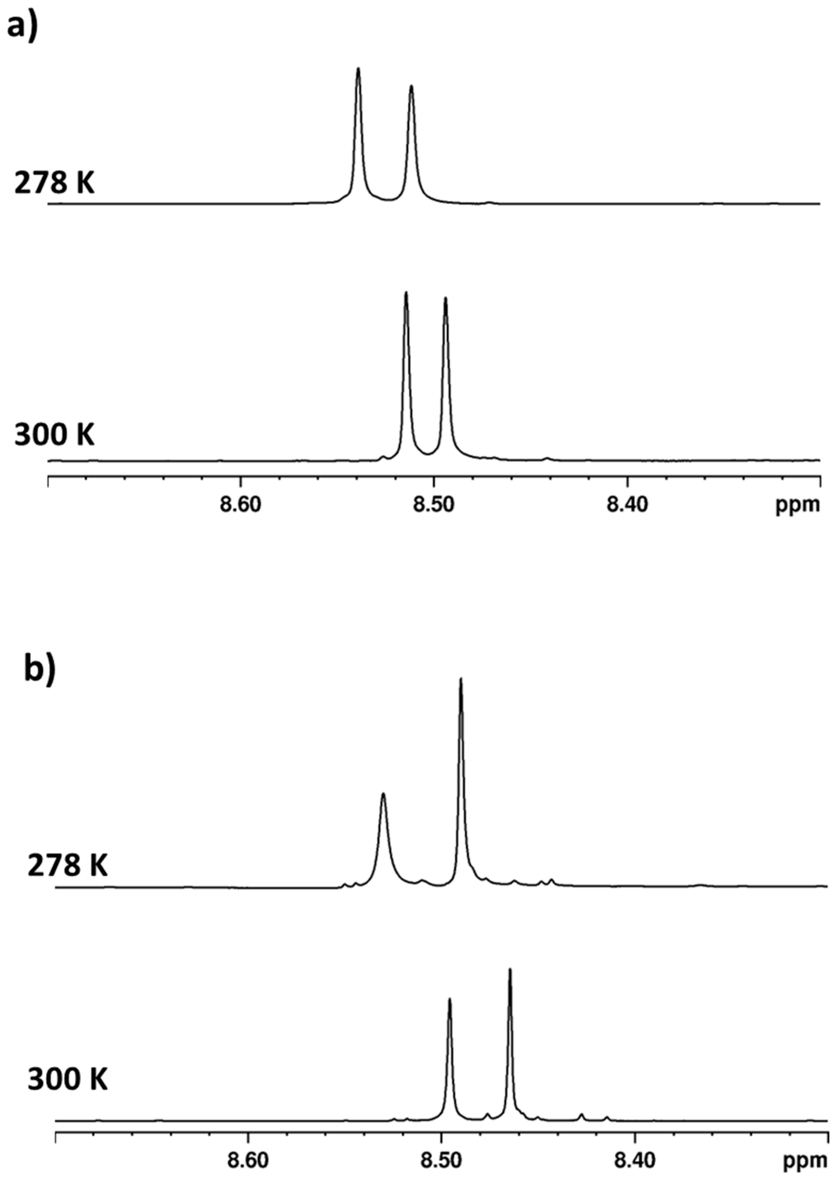

Figure S5: ^{1}H NMR spectra in the region of H8 resonances for the bis-adducts (*cis*-1,3-DACH)Pt(5'GMP)$_2$ (**a**) and (*cis*-1,3-DACH)Pt(3'GMP)$_2$ (**b**). Solvent D_2O/CD_3OD (2:1, v/v), pH* 5.83, temperatures 300 and 278 K.
